# German approach of coding rare diseases with ICD-10-GM and Orpha numbers in routine settings

**DOI:** 10.1186/1750-1172-9-S1-O10

**Published:** 2014-11-11

**Authors:** Stefanie Weber, Magdalena Dávila

**Affiliations:** 1 German Institute of Medical Documentation and Information, Cologne, Germany

## 

In Germany all physicians are compelled to code according to the International Statistical Classification of Diseases and Related Health Problems, 10th revision, German Modification (ICD-10-GM), according to the Statutory Health Insurance. The classification is in use since 2004 in the same Version for inpatient and outpatient care. Special coding guidelines are to be used for inpatient care.

DIMDI provides an Index to the ICD-10-GM, which contains approx. 80.000 terms. For electronic use the index is published with a unique identifier (so called Alpha-ID). This enables electronic communication of diagnosis on a more granular level than the broad categories of ICD-10.

In the German project “Coding of Rare diseases” (July 2013 – June 2016) all rare diseases from Orphanet will be coded according to ICD-10-GM and missing terms will be added to the ICD-10-GM-Index-Database. On a regular basis an Alpha-ID file with all indexed diagnostic terms together with the relevant codes from both systems will be published. Coders in Rare diseases Centers in Germany will be encouraged to use the two coding systems by implementing the file in their IT-systems. An evaluation of possible additional use of the file in existing IT-systems will be performed at the end of the project. The file could e.g. help to enable easy access to Orphanet for Users at the time of diagnosis, treatment, or when advising patient/family or coding from routine IT-applications in all kinds of healthcare settings.

Two main goals are targeted with the project. First of all it is important to standardize coding of rare diseases in Germany by providing electronic files for easy implementation, which will result in standardized code pairs from the two systems. Selecting codes manually from the two different systems is a potential for errors in patient documentation and might lead to incorrect statistical evaluation of rare disease cases. Secondly the aim of DIMDI is to reduce the administrative burden on the health care system in Germany whenever possible. By providing two coding systems in one file the coder will have to perform the coding just once, a significant reduction of time.

